# Surface properties-dependent antifungal activity of silver nanoparticles

**DOI:** 10.1038/s41598-022-22659-2

**Published:** 2022-10-27

**Authors:** Ewelina Matras, Anna Gorczyca, Sebastian Wojciech Przemieniecki, Magdalena Oćwieja

**Affiliations:** 1 Department of Microbiology and Biomonitoring, Faculty of Agriculture and Economics, University of Agriculture in Kraków, Mickiewicz Ave. 21, 31-120 Kraków, Poland; 2 Department of Entomology, Phytopathology and Molecular Diagnostics, University of Warmia and Mazury in Olsztyn, Prawocheńskiego 17, 10-720 Olsztyn, Poland; 3 Jerzy Haber Institute of Catalysis and Surface Chemistry, Polish Academy of Sciences, Niezapominajek 8, 30-239 Kraków, Poland

**Keywords:** Microbiology, Nanoscience and technology

## Abstract

Silver nanoparticles (AgNPs) exhibit unusual biocidal properties thanks to which they find a wide range of applications in diverse fields of science and industry. Numerous research studies have been devoted to the bactericidal properties of AgNPs while less attention has been focused on their fungicidal activity. Our studies were therefore oriented toward determining the impact of AgNPs characterized by different physicochemical properties on *Fusarium avenaceum* and *Fusarium equiseti*. The main hypothesis assumed that the fungicidal properties of AgNPs characterized by comparable morphology can be shaped by stabilizing agent molecules adsorbed on nanoparticle surfaces. Two types of AgNPs were prepared by the reduction of silver ions with sodium borohydride (SB) in the presence of trisodium citrate (TC) or cysteamine hydrochloride (CH). Both types of AgNPs exhibited a quasi-spherical shape. Citrate-stabilized AgNPs (TCSB-AgNPs) of an average size of 15 ± 4 nm were negatively charged. Smaller (12 ± 4 nm), cysteamine-capped AgNPs (CHSB-AgNPs) were characterized by a positive surface charge and higher silver ion release profile. The phytopathogens were exposed to the AgNPs in three doses equal to 2.5, 5 and 10 mg L^−1^ over 24 and 240 h. Additionally, the impact of silver ions delivered in the form of silver nitrate and the stabilizing agents of AgNPs on the fungi was also investigated. The response of phytopathogens to these treatments was evaluated by determining mycelial growth, sporulation and changes in the cell morphology. The results of our studies showed that CHSB-AgNPs, especially at a concentration of 10 mg L^−1^, strongly limited the vegetative mycelium growth of both species for short and long treatment times. The cell imaging revealed that CHSB-AgNPs damaged the conidia membranes and penetrated into the cells, while TCSB-AgNPs were deposited on their surface. The fungistatic (lethal) effect was demonstrated only for silver ions at the highest concentration for the *F. equiseti* species in the 240 h treatment. The number of spores of both *Fusarium* species was significantly reduced independently of the type of silver compounds used. Generally, it was found that the positively charged CHSB-AgNPs were more fungicidal than negatively charged TCSB-AgNPs. Thereby, it was established that the stabilizing agents of AgNPs and surface charge play a crucial role in the shaping of their fungicidal properties.

## Introduction

Agricultural production is under constant threat from various plant pathogens^1^. Despite the significant increase in the use of pesticides, losses caused by pathogens remain high. Globally, losses are estimated at 21.5% for wheat, 22.5% for maize and 30.0% for rice^2^. The dominant group of pathogens in cereal crops is fungi, which are responsible for approximately 80% of plant infections^3^. In 1991, it was estimated that there were 1.5 million species of fungi on Earth, but, at that time, only 70,000 fungi were well-identified taxonomically, making it an area ripe for investigation as the subject of further research^4^. New estimates suggest that there are 6.2 million species of fungi, confirming that fungi are ubiquitous^5^. *Fusarium* fungi are considered to be one of the most toxinogenic microorganisms in the world. They are able to change metabolism and adapt to the substrate on which they live, which makes it easier for them to quickly infect many species of plants and cereals that are important for human and animal nutrition^6^. The occurrence of these fungi is influenced by several factors, including agronomic practices and climatic conditions that cannot be controlled^7,8^. These factors affect the growth, survival and spread of the pathogen and thus the severity of the disease. For growth and development, most fungi prefer growing seasons of higher temperatures and high humidity^9,10^. Plants are infected at various stages of development, which causes a number of pre- and post-emergence diseases, such as Fusarium foot rot, Fusarium leaf blotch and Fusarium head blight^11–13^. Fungal infections reduce yields and lower the commercial quality of grain^14,15^. *Fusarium* fungi can cause diseases individually or in complex infections, which greatly complicate their control^6^. In addition, many species of the genus *Fusarium* have the potential to produce secondary metabolites known as mycotoxins that are toxic to humans, animals and plants^16,17^. Contamination of cereals with toxic metabolites of fungi is one of the most serious problems in world agriculture, as evidenced by numerous literature references and reports from institutions, i.e. the World Health Organization (WHO), the Joint FAO/WHO Expert Committee on Food Additives (JECFA) and the European Food Safety Authority (EFSA). According to the Food and Agriculture Organization (FAO), about 25% of global cereal production annually is contaminated with mycotoxins, although new data indicates that the global incidence of mycotoxins in crops is much higher (60–80%)^18^. Therefore, it is necessary to monitor the presence of these compounds mainly in food products. In 2007, the European Commission introduced unified standards regarding the acceptable level of mycotoxins in food^19^. In accordance with the legal regulations in force, exceeding the permissible concentrations of secondary metabolites excludes agricultural produce, fodder and food products from trade, causing huge economic losses.

Highly effective, modern and commercially available fungicides have revolutionized agriculture, especially in the case of plant pathogens which are difficult to control^20^. Despite their undoubted benefits, the massive use of conventional fungicides poses a risk to the environment and food safety, which is why an increasing number of active ingredients in fungicides are being phased out in line with EU regulations for the sake of safety^21^. For this reason, farmers are looking for alternative replacements for these products to eliminate or reduce plant pathogens^22^. Nanoparticles (NPs) could play an important role in solving this problem^23^. Silver nanoparticles (AgNPs) are one of the most abundant nanomaterials. The interest in these nanometric particles in agriculture is mainly due to their biocidal activity^20,24–27^. Silver is effective against 650 different microorganisms, and can therefore be used in the plant protection sector^28^. Silver exhibits oligodynamic activity against many cellular targets, unlike antibiotics which are selective^29,30^. AgNPs tend to attack many biological organelles, including the structure of the cell membrane, as a result of which electron transport is interrupted and cellular metabolism is ultimately disrupted^31,32^. In addition, AgNPs damage DNA, inhibit protein synthesis related to the production of ATP, and can generate the production of reactive oxygen species (ROS), which causes oxidative stress and, as a result, inhibits cell proliferation^33–36^.

The available literature shows that the biocidal activity of AgNPs is mainly determined by their size, surface properties and the concentration used^37–39^. Smaller AgNPs have been found to be more toxic than larger ones^40^. However, the presence of stabilizers adsorbed on the AgNP surface seems to be the main factor modeling biological activity^41,42^. The appropriate chemical structure of stabilizing agents enables the control of the electrokinetic properties of AgNPs. Most frequently, AgNPs stabilized with inorganic anions are negatively charged, while those coated with organic compounds having groups capable of protonating and deprotonating may be negatively or positively charged^43^. In addition, stabilizing agents can induce the oxidative dissolution of AgNPs, which causes the release of silver ions that interact with various biomolecules within the cell, such as nucleic acids, cell wall components, thiol groups (-SH) of the cysteine amino acid, which is a component of structural and enzymatic proteins, and compounds containing sulfur and phosphorus^35,36^. Moreover, biologically active chemicals used as stabilizers can intensify the penetration of AgNPs through biological membranes and facilitate their accumulation in cell organelles^44^.

There are many studies in the literature on the fungitoxic activity of AgNPs, while very little data is available on the effect of AgNPs surface properties on mycelium growth and sporulation of plant pathogens. Therefore, the aim of our study was to evaluate the 24 and 240 h treatment with AgNPs characterized by various surface properties, against the background of silver ions delivered in the form of AgNO_3_ against *F. avenaceum* and *F. equiseti* under in vitro conditions. It was assumed that the biological activity of AgNPs would depend on the properties of the stabilizer molecules adsorbed on their surface and the surface charge generated by these molecules. Assessment of the reaction of fungal conidia to silver compounds will provide pivotal information on their potential use as plant protection products, as the methods of controlling the toxinogenic fungi of the genus *Fusarium* are still imperfect. The obtained results will also be important for further research into the potential of AgNPs.

## Materials and methods

### Reagents

Silver nitrate (AgNO_3_), trisodium citrate dihydrate (TC), sodium borohydride (SB), cysteamine hydrochloride (CH) and ammonia solution (25%) were purchased from Sigma Aldrich. These reagents were used without further purification as they were of analytical grade. Ultrapure water used for preparation of the AgNP suspensions was produced by a Milli-Q Elix & Simplicity 185 purification system (Millipore SA Molsheim).

Two types of AgNPs were used in the study: AgNPs obtained with the use of sodium borohydride (SB) in the presence of trisodium citrate (TC) (marked as TCSB-AgNPs) and AgNPs synthesized applying sodium borohydride (SB) and cysteamine hydrochloride (CH) (marked as CHSB-AgNPs). The preparation procedures of both suspensions and detailed physicochemical characteristics of AgNPs were described in the previous work^45^.

### Biological material

Two species of fungi of the genus *Fusarium* were used for the study: *F. avenaceum* (Fries) Saccardo (strain designation: M 48.1) and *F. equiseti* (Corda) Sacc (strain designation: M 55.2), which are of great importance as toxinogenic pathogens in cereal crops. These species were isolated from wheat grown near Kraków (Southern Poland, 50°06′52″N, 20°04′23″E) in 2016. The isolates were molecularly determined at the Institute of Plant Genetics, Polish Academy of Sciences in Poznań. The pure fungal cultures obtained were passaged onto standard Potato Dextrose Agar (PDA) (Biocorp) to obtain sporulating mycelium. Conidia suspensions of fungi prepared from fresh cultures on PDA medium were treated. Spore suspensions were prepared by introducing fresh fungi culture into 300 mL glass bottles with sterilized water (100 mL). The bottles were shaken for 1 h and the solution was sterile-filtered to obtain only the spore cells in water. Conidia concentration was assessed using a Bürker counting chamber. The conidia suspensions of both *Fusarium* species tested were adjusted by the dilution method to a concentration of 5·10^7^ conidia mL^−1^,which was the initial test concentration.

### Experimental part

#### Experimental methods to determine AgNP characteristics

A DMA500M densitometer was used to measure the density of purified AgNP suspensions and effluents obtained during the purification procedure^45^. Then, the mass concentration of AgNPs in the stock suspensions was calculated based on the density measurements^45^. A JEOL JSM-7500F electron microscope was used to record micrographs of AgNPs and evaluate their morphology and size distribution. The histograms were prepared using MultiScan software^45^. A Zetasizer Nano ZS instrument was used to determine the diffusion coefficients and electrophoretic mobility of AgNPs dispersed in the suspensions of controlled pH, ionic strength and temperature. The concentration of silver ions leached from the AgNPs was determined using a PinAAcle 900Z atomic absorption spectrometer (AAS). For this purpose, the measurement protocol described previously^43,45^ was employed.

#### Linear growth and growth rate index of mycelium

The treatments were performed in vitro by shaking the aqueous solutions of the conidia of the fungal species with the AgNP suspensions and silver ions of controlled mass concentration in a Biosan ES-20/60 shaker, in the volume ratio of 1:1 at a constant temperature of 21°C in 100 mL Erlenmeyer flasks. The control variant was carried out for the spores of both fungi species in sterilized water. Additional investigations were conducted for the spores exposed to the solutions of both types of stabilizing agents of AgNPs, namely TC and CH. The concentration of stabilizing agents in the aqueous solutions was equal to 10 mg L^−1^. The impact of both types of AgNPs, silver ions, as well as TC and CH on the spores was assessed after 24 and 240 h of treatment. For growth evaluation, treated conidia suspensions were immediately pipetted in a volume of 100 µL onto PDA medium in 5 replicates for each variant. The Petri dishes were incubated in a controlled atmosphere (dark, 21°C) Biogenet MDF 500 growth chamber. The diameter of mycelial growth was measured. The average mycelial growth rate index in the culture [mm h^−1^], which lasted 168 h, was also calculated using measurements of the mycelium diameter performed regularly every 12 h.

#### Mycelium sporulation

The spore formation of the fungal cultures was assessed after 24 and 240 h of incubation of the fungi treated with the both types of AgNPs, their stabilizing agents (TC and CH) and silver ions, and grown on PDA. Spore-forming mycelial discs with a diameter of 10 mm were placed in 100 mL Erlenmeyer flasks containing 10 mL of sterilized water supplemented with Tween 80 (Sigma-Aldrich). The flasks were shaken for 1 h and the suspension was filtered under sterile conditions. The spore concentration was determined using a Bürker counting chamber. 5 repetitions were performed for each variant. A microscopic image of the chamber was photographed with a Moticam 1000 (Motic) and the resulting images were analyzed using ImageTool (University of Texas Health Science Center in San Antonio).

#### Transmission electron microscope (TEM) conidia image

TEM was used to examine the ultrastructure of treated and untreated fungal cells. TEM imaging of treated conidia for *F. avenaceum* species was performed with a JEOL JEM2100 HT CRYO LaB6 microscope after 240 h of treatment.

### Statistical analyses

The data was first evaluated for normality of distribution (Shapiro–Wilk test) and homogeneity of variance (Levene’s test), then the differences between results were determined by analysis by a Kruskal–Wallis test at a significance determined by Dunn’s test with Bonferroni correction. The relationships between observations were determined by Principal Component Analysis (PCA) based on Pearson’s correlation matrix, and Agglomerative Hierarchical Clustering (AHC) based on Bray and Curtis dissimilarity with Ward’s method. To construct the Pearson correlation matrix for PCA, we used the centralization of the results for each trait according to the formula: ((result-mean)/standard deviation). The Spearman's Rank Correlation was used to discover the strength of a link between the growths of *Fusarium* spp. mycelium after treatments with compounds. The results were processed statistically in XLSTAT software (Addinsoft, UK).

## Results

### Physicochemical characteristics of AgNPs

Based on the TEM micrographs, it was established that TCSB-AgNPs and CHSB-AgNPs exhibited a quasi-spherical shape and comparable size distribution. Typical TEM micrographs and the size distribution of AgNPs (determined based on analysis of these images^45^) are presented in the Supplementary materials (Fig. S1). The average size of TCSB-AgNPs and CHSB-AgNPs was equal to 15 ± 4 nm and 12 ± 4 nm, respectively (cf. the Supplementary materials, Table S1). In turn, the hydrodynamic diameters of TCSB-AgNPs and CHSB-AgNPs, calculated from the measurements of diffusion coefficients (cf. the Supplementary materials, Table S1) attained values of 16 ± 5 nm and 13 ± 3 nm, respectively. Thereby, the average sizes of AgNPs determined from the TEM imaging and the measurements conducted with the use of the Dynamic Light Scattering (DLS) technique remained in good agreement.

The measurements of electrophoretic mobility revealed that TCSB-AgNPs were negatively charged, whereas CHSB-AgNPs were characterized by positive surface charge (the Supporting materials, Table S1). The zeta potential of TCSB-AgNPs attained a value of − 66 ± 3 mV, while CHSB-AgNP zeta potential was equal to + 58 ± 2 mV at a pH of 5.8 (unregulated ionic strength, measurements conducted for the AgNPs dispersed in the stock suspensions). It was found that negatively charged TCSB-AgNPs were less prone to oxidative dissolution than CHSB-AgNPs (cf. the Supporting materials, Table S1).

### Linear growth and growth rate index of mycelium

**Figure 1:**
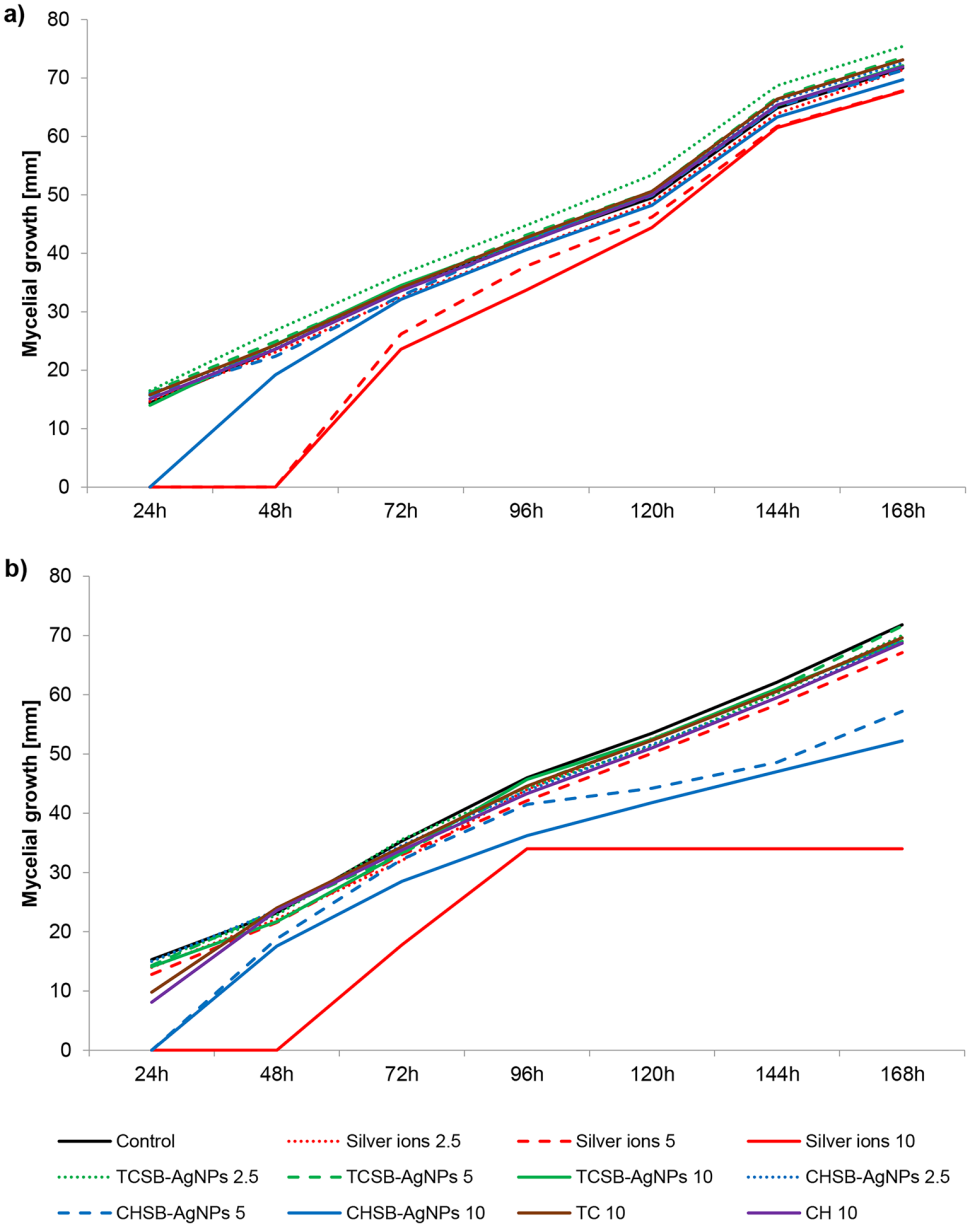
The impact of silver ions, AgNPs and stabilizing agents on the mycelial growth of *F. avenaceum* over 24 h (**a**) and 240 h (**b**) of the exposure period. The number given for each treatment represents the concentration expressed in mg L^−1^.

Figure 1a and Table S2 (Supporting materials) show the effect of silver compounds and the AgNP stabilizing agents on the growth of *F. avenaceum* in a 24 h treatment. Brief (24 h) exposure of conidia to silver ions at concentrations of 5 and 10 mg L^−1^ resulted in a significant reduction in the growth of *F. avenaceum*. These solutions of silver ions were fungistatic for the conidia of the fungus in the 24 and 48 h culture. CHSB-AgNPs applied at the highest concentration also inhibited the growth in the 24 h culture and significantly limited the growth of this fungus in the 48 h culture. In the following hours of cultivation, lower values of mycelial growth were observed than in the control, but it was not statistically significant. It is worth noting that the greatest diameter of *F. avenaceum* mycelium occurred for the conidia treated with TCSB-AgNPs at concentrations of 2.5 and 5 mg L^−1^ (Fig. 1a, Table S2a Supporting materials).

In the case of longer treatment of *F. avenaceum* conidia (240 h), a delay in colony formation was observed after the application of silver ions at the highest concentration (Fig. 1b). The same relationship was observed for the CHSB-AgNP treatment at concentrations of 5 and 10 mg L^−1^ (Fig. 1b, Table S2b Supporting materials). Treatment with silver ions at the concentration of 10 mg L^−1^ and CHSB-AgNPs at concentrations of 5 and 10 mg L^−1^ resulted in what was significantly the greatest reduction in the growth of *F. avenaceum*. Significantly weaker growth of conidia compared to the control was also found for the samples exposed to the action of CH. The other compounds, including TC and TCSB-AgNPs, did not differ statistically significantly from the control (Fig. 1b).

**Figure 2:**
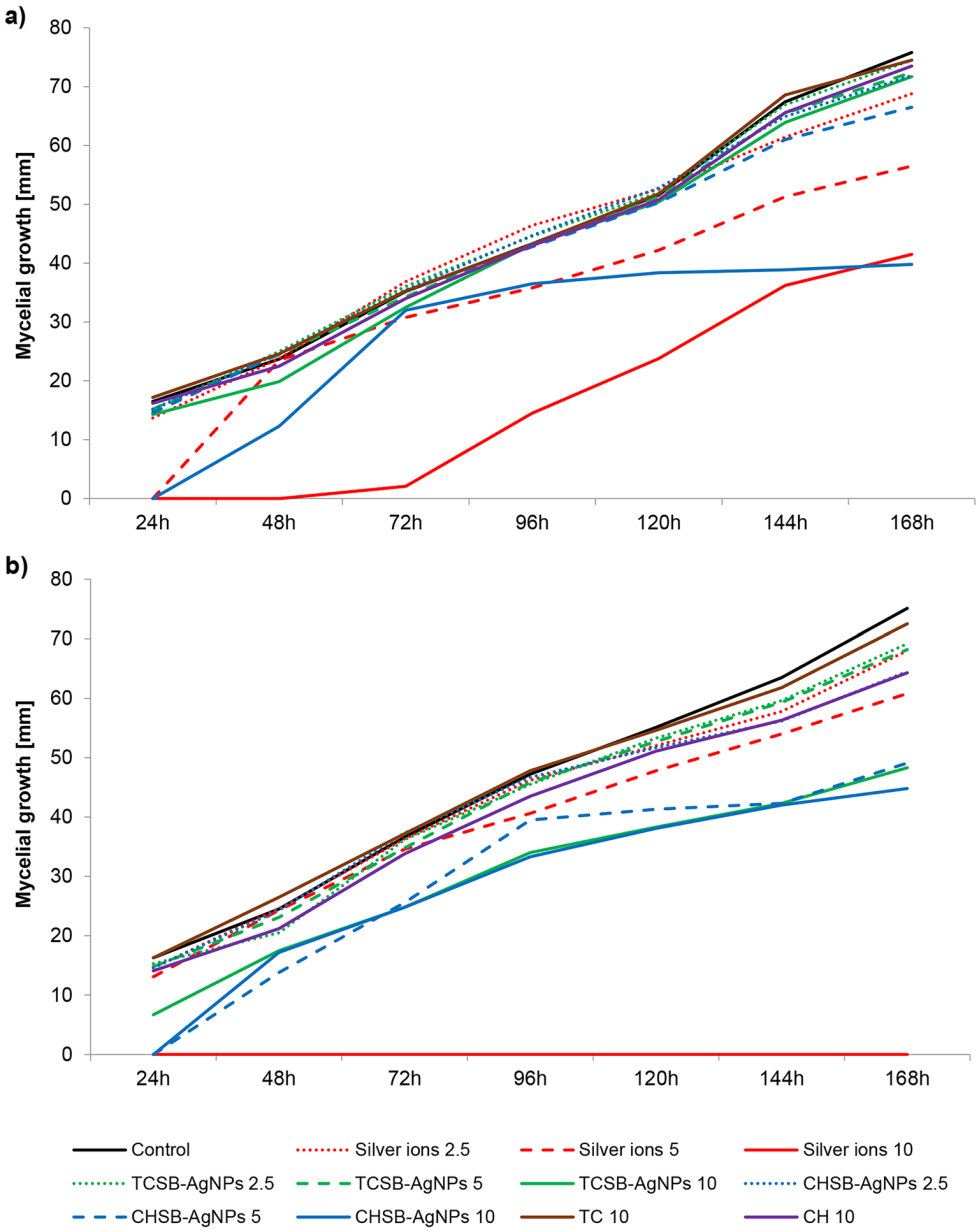
The impact of silver ions, AgNPs and stabilizing agents on the mycelial growth of *F. equiseti* over 24 h (**a**) and 240 h (**b**) of the exposure period. The number given for each treatment represents the concentration expressed in mg L^−1^.

Figure 2a and Table S3a (Supporting materials) present the data obtained for *F. equiseti* mycelium during the 24 h of treatment with the silver compounds and the AgNP stabilizing agents. The greatest limitation of growth was observed after contact of the conidia with silver ions at concentrations of 5 and 10 mg L^−1^ and CHSB-AgNPs at the highest concentration. In the case of the treatments with the use of TCSB-AgNPs and the stabilizing agents (CH, TC), negligible differences in conidia growth were observed in comparison to the control group (Fig. 2a, Table S3, Supporting materials).

Based on the results of studies obtained for the 240-h exposure to the used compounds, it was established that the *F. equiseti* species turned out to be more sensitive to the AgNPs, silver ions and stabilizing agents than in the short treatment (Fig. 2b, Table S3b Supporting materials). The *F. equiseti* conidia were completely inactivated after the treatment with the silver ions at the highest concentration. Moreover, silver ions at a concentration of 5 mg L^−1^, CHSB-AgNPs at concentrations of 5 and 10 mg L^−1^, TSCB-AgNPs at a concentration of 10 mg L^−1^, and CH at a concentration of 10 mg L^−1^ significantly limited the growth of *F. equiseti*.

Based on the Spearman rank correlation for the growth of *Fusarium* spp. mycelium after the treatments with the silver ions, AgNPs and AgNP stabilizing agents, a very strong relationship was found between *F. avenaceum* and *F. equiseti* (r = 0.920, p < 0.0001). It was established that the response of both fungi to the investigated compounds was almost identical (Table S4, Supporting materials).

**Figure 3:**
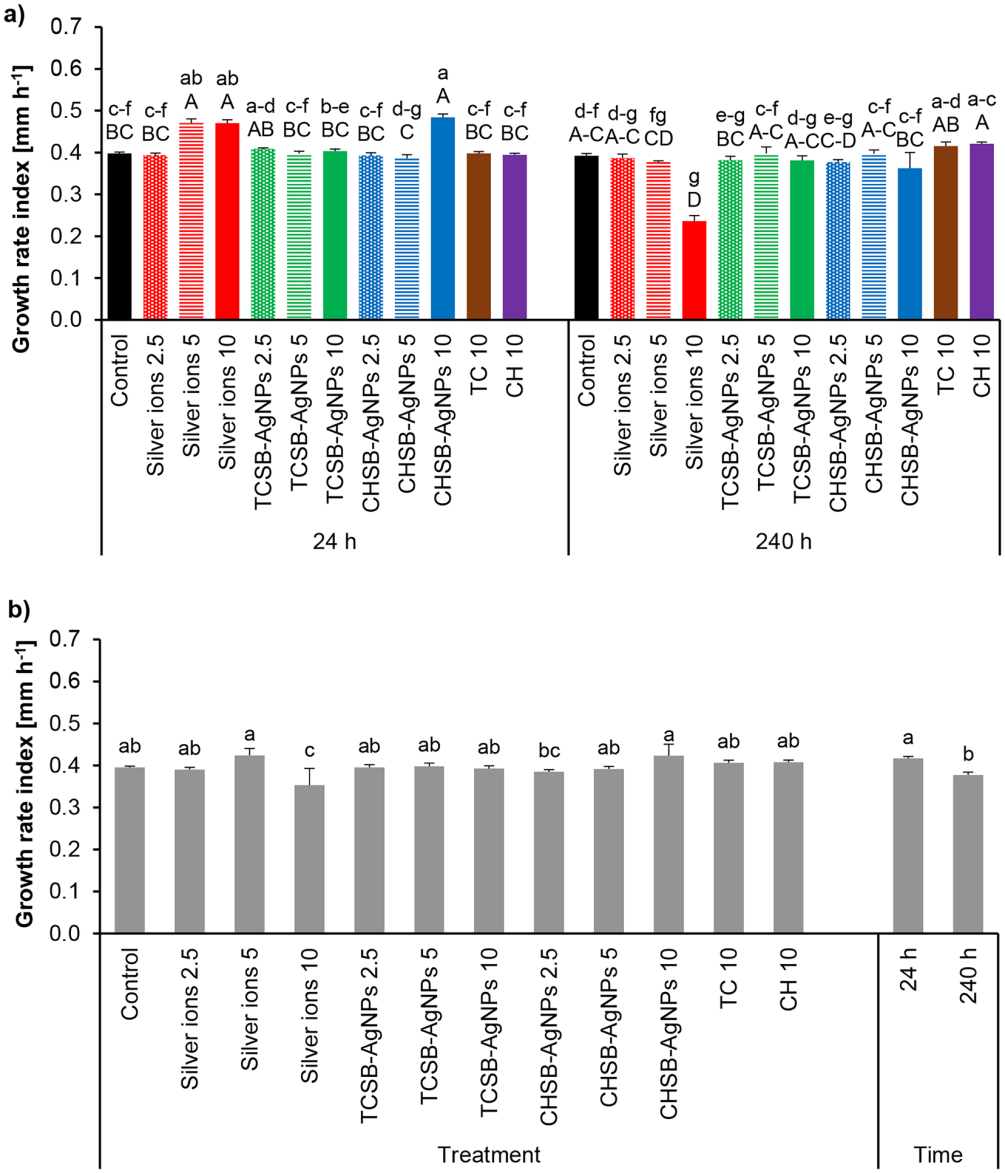
The impact of silver ions, AgNPs and stabilizing agents on the mycelium growth rate index of *F. avenaceum* after culture over 24 h and 240 h of the exposure period: factors interaction effect (a), factors effect (b). Data represents mean values ± SE. Values marked with the same letters (lowercase letters for the interaction effect, as well as the main effects of time and treatment; capital letters for interaction effect separately for the hours) are not significantly different at p ≤ 0.05. The number given for each treatment represents the concentration expressed in mg L^−1^.

Figure 3a shows the effect of silver ions, AgNPs and stabilizing agents of AgNPs on the mycelium growth rate index of *F. avenaceum* after the end of exposure to the compounds lasting 24 and 240 h. In the case of a short treatment time (24 h), the highest indexes of the mycelial growth rate were obtained after the application of CHSB-AgNPs at a concentration of 10 mg L^−1^, as well as silver ions at concentrations of 5 and 10 mg L^−1^. The total inhibition of growth or weaker growth in these variants and the subsequent faster growth rate of the mycelium can be explained by their response to the stress conditions. In contrast, the treatment of *F. avenaceum* conidia for 240 h resulted in a lower growth rate only for silver ions at a concentration of 10 mg L^−1^ compared to control. An analysis of the main factors showed that *F. avenaceum* treated with the investigated compounds for 240 h showed a lower growth rate than in the case of the short treatment (Fig. 3b). Of the treatments used, only the highest concentration of silver ions significantly reduced the growth rate of *F. avenaceum* (Fig. 3b). The remaining compounds did not differ significantly from the controls.

**Figure 4:**
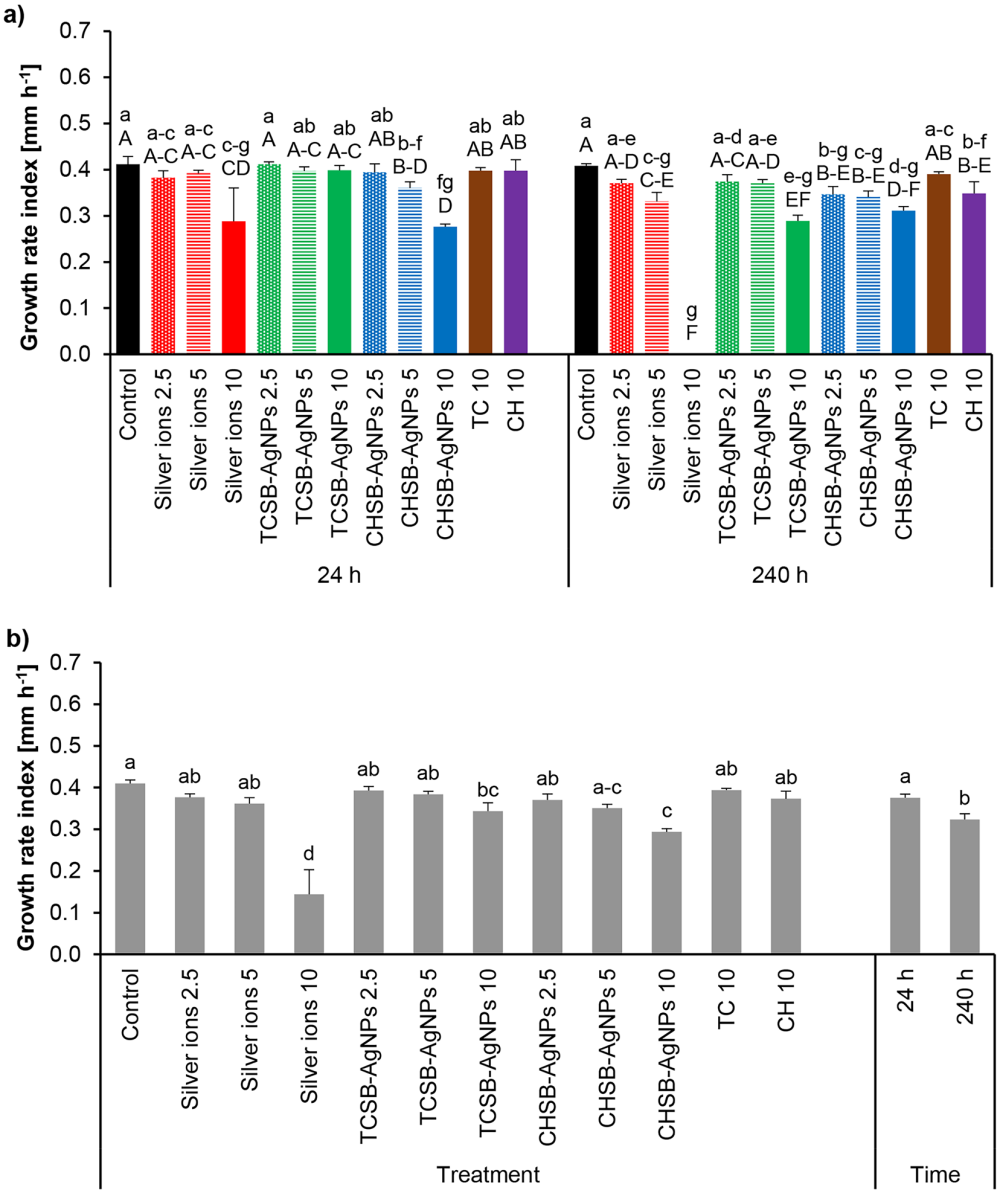
The impact of silver ions, AgNPs and stabilizing agents on the mycelium growth rate index of *F. equiseti* after culture over 24 h and 240 h of the exposure period: factors interaction effect (**a**), factors effect (**b**). Data represents mean values ± SE. Values marked with the same letters (lowercase letters for the interaction effect, as well as the main effects of time and treatment; capital letters for interaction effect separately for the hours) are not significantly different at p ≤ 0.05. The number given for each treatment represents the concentration expressed in mg L^−1^.

The influence of the investigated experimental factors on the sporulation of *F. equiseti* is shown in Fig. 4a. The lowest index of the growth rate of *F. equiseti* mycelium was obtained after the 24 h treatment of conidia with CHSB-AgNPs at concentrations of 5 and 10 mg L^−1^ and silver ions at the highest concentration. Longer contact (240 h) of conidia with silver ions at a concentration of 10 mg L^−1^ resulted in complete inhibition of the growth of *F. equiseti* mycelium (Fig. 4a). In addition, silver ions at a concentration of 5 mg L^−1^, TCSB-AgNPs at the highest concentration, CHSB-AgNPs at concentrations of 5 and 10 mg L^−1^ and CH at a concentration of 10 mg L^−1^ also significantly reduced the mycelial growth rate compared to the control. An analysis of the main factors showed that longer treatment of *F. equiseti* conidia with the investigated compounds reduced the mycelial growth rate more than short treatment (Fig. 4b). The lowest growth rate was noted for the treatment with the use of silver ions at a concentration of 10 mg L^−1^, followed by CHSB-AgNPs at concentrations of 5 and 10 mg L^−1^ and TCSB-AgNPs at the highest concentration only (Fig. 4b).

### Mycelium sporulation

**Figure 5:**
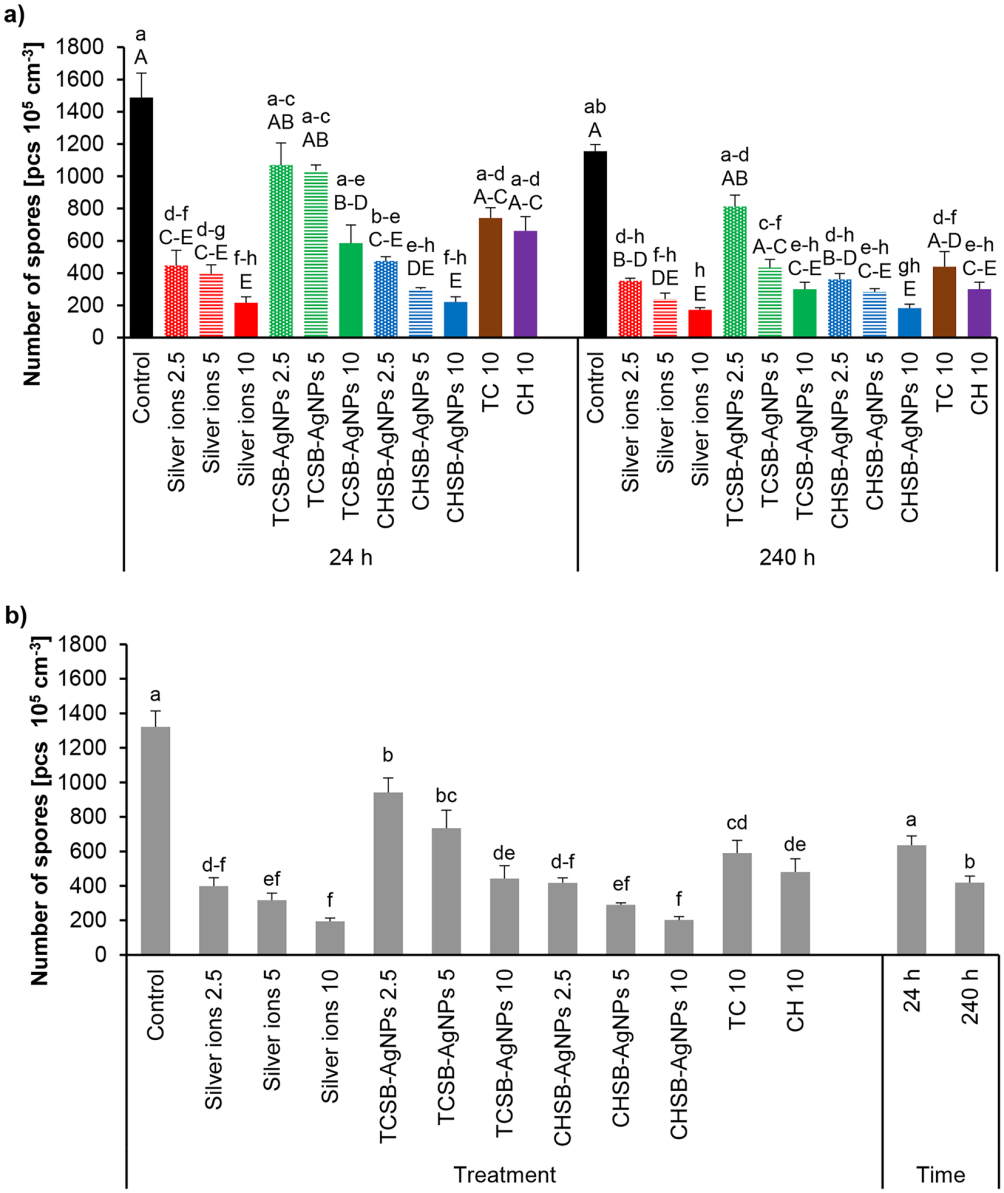
The impact of silver ions, AgNPs and stabilizing agents on the sporulation of *F. avenaceum* after culture over 24 h and 240 h of the exposure period: factors interaction effect (**a**), factors effect (**b**). Data represents mean values ± SE. Values marked with the same letters (lowercase letters for the interaction effect, as well as the main effects of time and treatment; capital letters for interaction effect separately for the hours) are not significantly different at p ≤ 0.05. The number given for each treatment represents the concentration expressed in mg L^−1^.

Figure 5a shows the interaction of the influence of the investigated experimental factors on *F. avenaceum* sporulation. 24 h contact of *F. avenaceum* with silver ions, CHSB-AgNPs at each investigated concentration and TCSB-AgNPs at the highest concentration resulted in a significant reduction of sporulation of the mycelium in comparison to the control group. In the case of silver ions and CHSB-AgNPs at the concentration of 10 mg L^−1^, seven times less spores were observed than in the control variant. As in the case of the short treatment, the same silver compounds and CH at a concentration of 10 mg L^−1^ significantly reduced sporulation of the fungus in the long treatment (240 h). An analysis of the main factors showed that confirmed that with the passage of time, sporulation of *F. avenaceum* was reduced to a greater extent, regardless of the type of the compound used (Fig. 5b). Considering the effect of the treatment alone on the sporulation of the *F. avenaceum* mycelium, it was found that all silver compounds and TC and CH significantly reduce this parameter (Fig. 5b).

**Figure 6:**
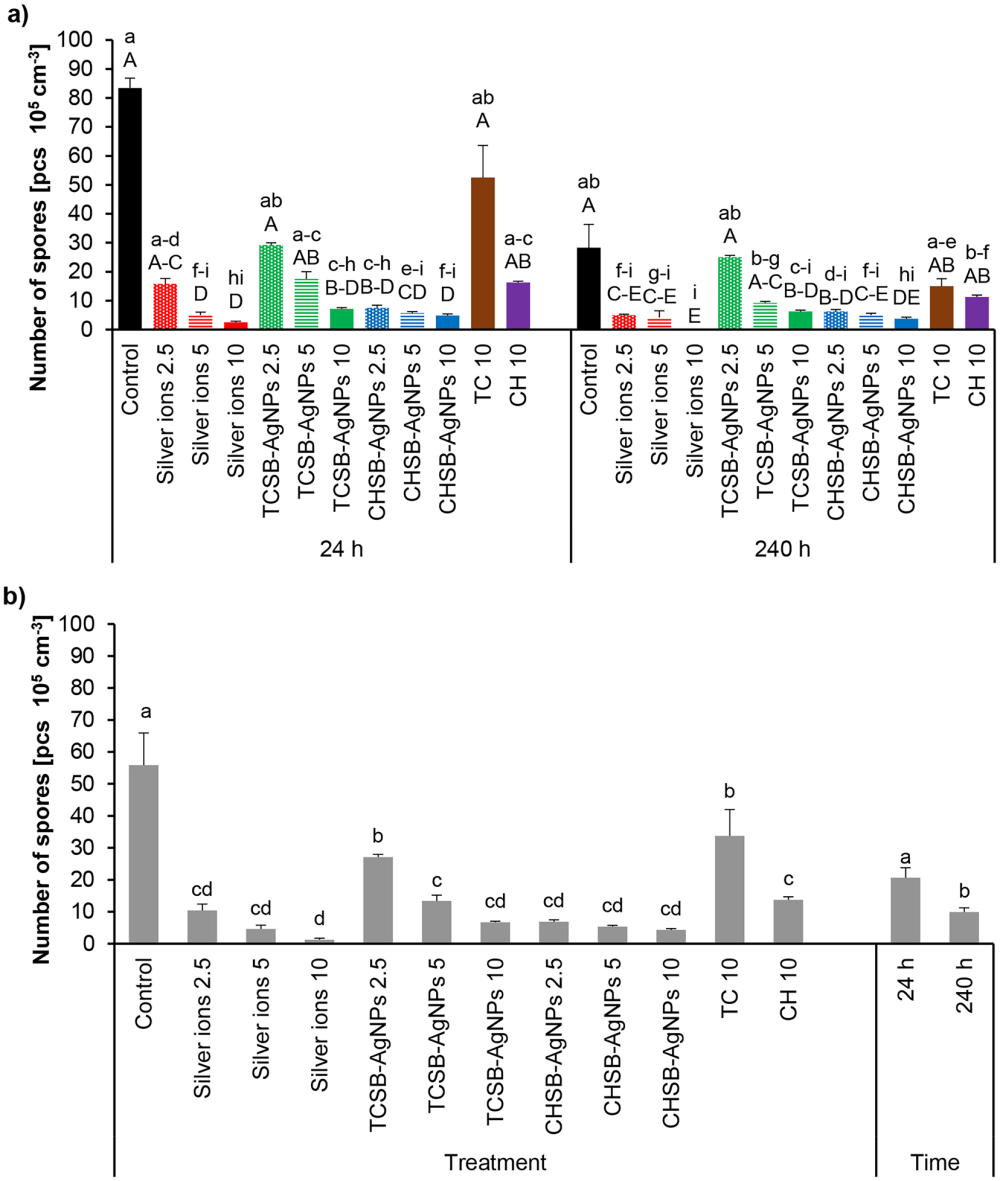
The impact of silver ions, AgNPs and stabilizing agents on the sporulation of *F. equiseti* after culture over 24 h and 240 h of the exposure period: factors interaction effect (**a**), factors effect (**b**). Data represents mean values ± SE. Values marked with the same letters (lowercase letters for the interaction effect, as well as the main effects of time and treatment; capital letters for interaction effect separately for the hours) are not significantly different at p ≤ 0.05. The number given for each treatment represents the concentration expressed in mg L^−1^.

The influence of the investigated experimental factors on the number of *F. equiseti* spores is shown in Fig. 6a. The number of spores after 24 h of the treatment with silver ions at the concentrations of 5 and 10 mg L^−1^, TCSB-AgNPs at the highest concentration and CHSB-AgNPs at each investigated concentration decreased significantly compared to the control group. Based on the results obtained, it was found that CHSB-AgNPs at the highest concentration and silver ions at the concentrations of 5 and 10 mg L^−1^ were the most toxic. In the case of 24-h treatment, silver ions and CHSB-AgNPs at each investigated concentration and TCSB-AgNPs at a concentration of 10 mg L^−1^ reduced sporulation compared to controls. *F. equiseti* did not form spores at all in case of silver ions at the highest concentration. As with *F. avenaceum* (Fig. 5b), an analysis of the main factors showed that longer contact of *F. equiseti* conidia with the investigated compounds resulted in greater reduction of sporulation than shorter treatment (Fig. 6b). In the case of second factor-treatment, all silver compounds and stabilizers were shown to be toxic to *F. equiseti* spores (Fig. 6b).

### Transmission electron microscope (TEM) conidia image

**Figure 7:**
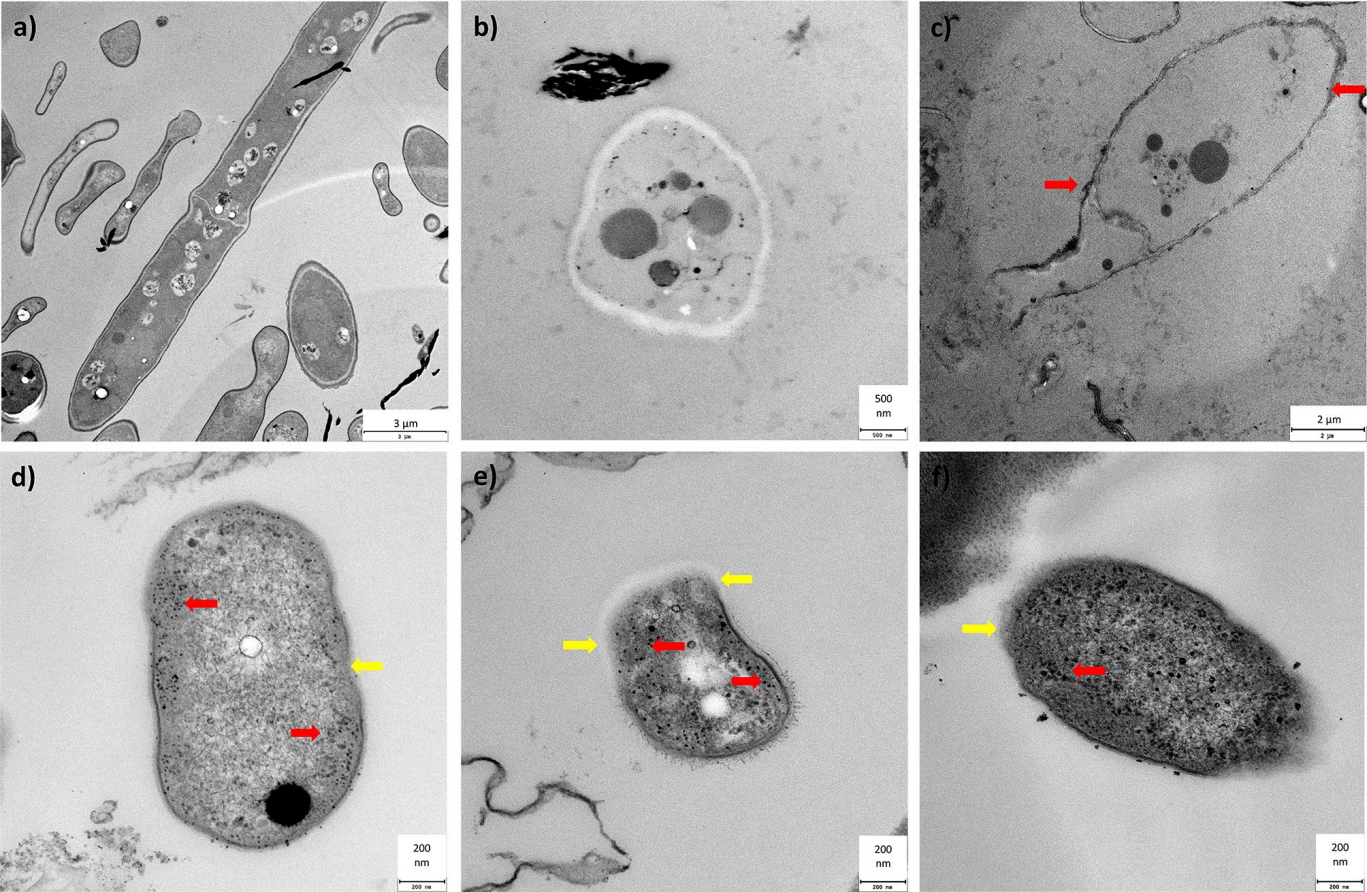
TEM micrographs of *F. avenaceum* conidia treated with water (control) (**a**), silver ions (**b**), TCSB-AgNPs (**c**), CHSB-AgNPs (**d**,**e,f**) at a concentration of 10 mg L^−1^ after culture for 240 h.

TEM was used to evaluate the ultrastructure of *F. avenaceum* cells, both in the control and those treated with silver compounds at a concentration of 10 mg L^−1^ after culture for 240 h (Fig. 7a–f). In the case of the control, the longitudinal section of the spores was presented, while for the silver ions and AgNPs – a transverse section was examined. As shown in Fig. 7a, the cell structure of the untreated cell was intact, with a clearly distinct cell wall. However, micrographs of conidia treated with both AgNPs showed different changes, confirming their antifungal activity. It was shown that TCSB-AgNPs at a concentration of 10 mg L^−1^ was deposited on the surface of the fungal cell wall (Fig. 7c, red arrows). CHSB-AgNPs at a concentration of 10 mg L^−1^ were more fungistatic than TCSB-AgNPs because they caused local disintegration of the cell wall (yellow arrows), which allowed them to easily penetrate inside the cells (red arrows) (Fig. 7d–f). After the damage to the membrane, the intracellular contents leaked and the internal structures were deformed (Fig. 7f).

### AHC and PCA analysis

**Figure 8:**
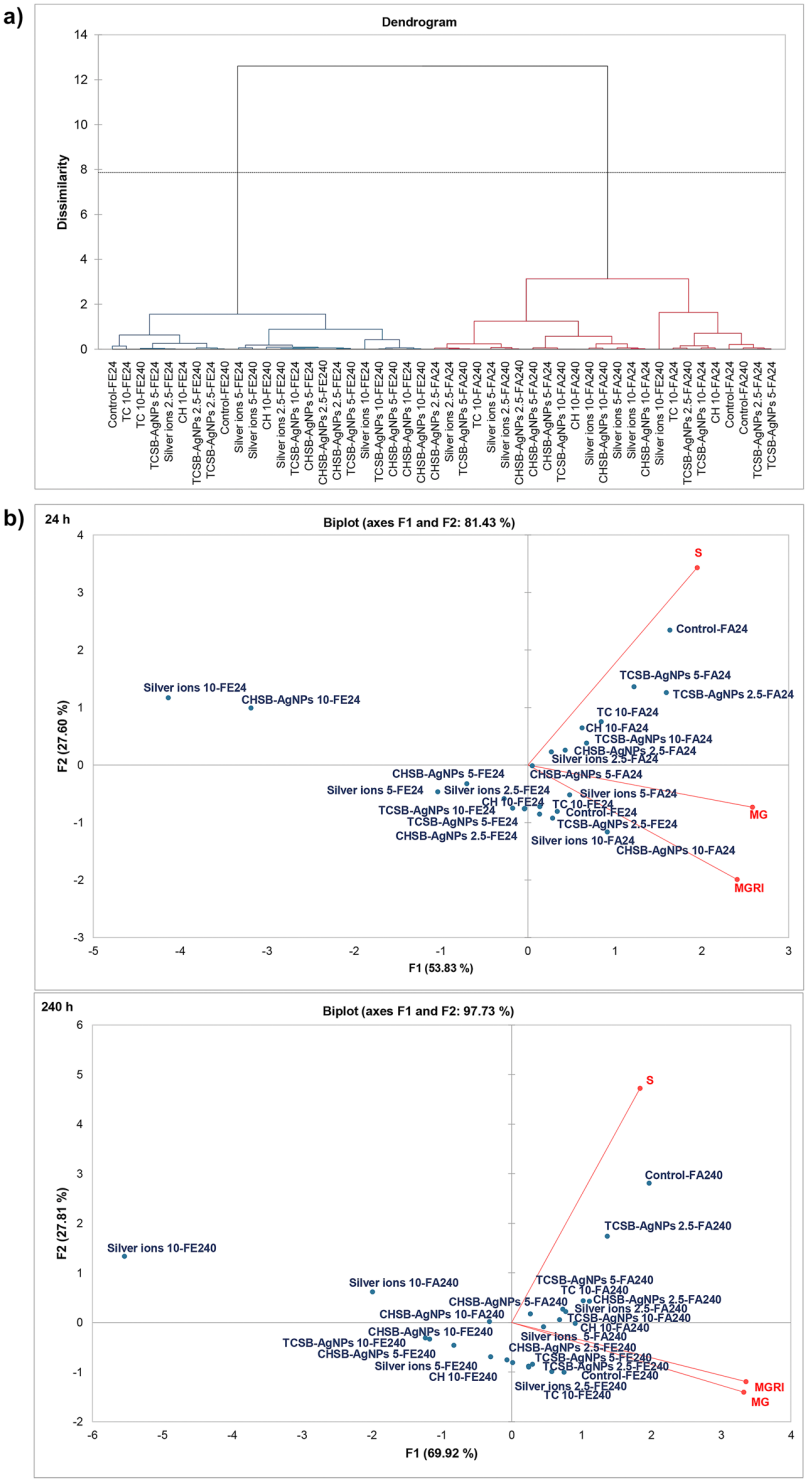
Interaction between silver ions, AgNPs and stabilizing agents, and fungi parameters over 24 h and 240 h on the basis of agglomerative hierarchical clustering (**a**) and principal component analysis (**b**). FA, *F. avenaceum*; FE, *F. equiseti*; S, sporulation; MG, mycelial growth; MGRI, mycelium growth rate index. The number given for each treatment represents the concentration expressed in mg L^−1^.

Based on the results of the hierarchical clustering of agglomerations (AHC), a clear grouping of the fungi into two clades consisting of *F. equiseti* and *F. avenaceum* was observed (Fig. 8a). When analyzing the arrangement of the AHC dendrogram for variant distribution, it was observed that, in the *F. equiseti* clade, two groups were formed, which in turn were divided into two subgroups consisting of both short and long treatment times. In the first subgroup (from the left), the effect of the applied measures, i.e. the controls and TC at the concentration of 10 mg L^−1^ (24 h), had a similar effect. The second subgroup consisted of the controls (240 h), TCSB-AgNPs at the concentrations of 2.5 (24 and 240 h) and 5 mg L^−1^ (24 h), silver ions at the lowest concentration (24 h) and the reagents—TC (240 h) and CH (24 h) at the concentration of 10 mg L^−1^. The second group of the *F. equiseti* clade was more extensive. The first subgroup consisted of silver ions at the concentration of 5 mg L^−1^ (24 and 240 h), silver ions at the concentration of 2.5 mg L^−1^ (240 h), CH (240 h), TCSB-AgNPs at the concentrations of 5 (240 h) and 10 mg L^−1^ (24 h), CHSB-AgNPs at concentrations of 2.5 (24 and 240 h) and 5 mg L^−1^ (24 h). The second subgroup consisted of increasing dissimilarity: CHSB-AgNPs at concentrations of 5 mg L^−1^ (240 h) and 10 mg L^−1^ (24 and 240 h), and TCSB-AgNPs at a concentration of 10 mg L^−1^ (240 h) < silver ions (24 h). The second clade of *F. avenaceum* also consisted of two groups. The first group consisted of 2 subgroups. The first subgroup consisted of CHSB-AgNPs and silver ions at the lowest concentration (24 h), TCSB-AgNPs at a concentration of 5 mg L^−1^ (240 h), TC (240 h) and silver ions at concentrations of 2.5 (240 h) and 5 mg L^−1^ (24 h), and CHSB-AgNPs at a concentration of 2.5 mg L^−1^ (240 h). The second subgroup included CHSB-AgNPs at the concentrations of 5 mg L^−1^ (24 and 240 h) and 10 mg L^−1^ (24 and 240 h), and TCSB-AgNPs at the highest concentration (240 h), CH (240 h) and silver ions at the concentrations of 5 mg L^−1^ (240 h) and 10 mg L^−1^ (24 and 240 h). The second group consisted of a first subgroup with little differentiation—silver ions at the highest concentration (240 h) and the second, more extensive subgroup. The second subgroup consisted of TC at the concentration of 10 mg L^−1^ (240 h), TCSB-AgNPs at the concentrations of 2.5 mg L^−1^ (24 and 240 h), 5 and 10 mg L^−1^ (24 h), CH at the concentration of 10 mg L^−1^ (24 h), and the controls (24 and 240 h).

The PCA biplot for the interaction between the compounds and the tested fungal parameters is shown separately for each treatment time (24 h and 240 h) in Fig. 8b. In each case, the first two factors (F1 and F2) show the high values of the initial data variability, i.e. 81.43% for the short treatment and 97.73% for the long treatment, which proves the effectiveness of the test performed. The effect of investigating compounds on fungi in the short treatment (24 h) showed that TCSB-AgNPs at concentrations of 2.5 and 5 mg L^−1^ had no global effect on sporulation, growth and the growth rate index in *F. avenaceum* compared to the control. It was found that TCSB-AgNPs at a concentration of 10 mg L^−1^, CHSB-AgNPs and silver ions at a concentration of 2.5 mg L^−1^ induced less of an effect on *F. avenaceum* sporulation. An intermediate impact on the sporulation process was noted in the case of CHSB-AgNPs at a concentration of 5 mg L^−1^. In turn, silver ions and CHSB-AgNPs at concentrations of 5 and 10 mg L^−1^ exhibited the strongest action. Moreover, silver ions at concentrations of 5 and 10 mg L^−1^ and CHSB-AgNPs at a concentration of 10 mg L^−1^ also inhibited mycelium growth the most, while they favored the mycelium growth rate index, which may be due to stressful conditions. In the case of *F. equiseti* also silver ions and CHSB-AgNPs at the highest concentration were the most fungistatic. CHSB-AgNPs and silver ions at a concentration of 5 mg L^−1^ also had a negative effect on the parameters, while the results for the remaining treatments were in close proximity to each other, suggesting a similar effect of these compounds.

The results of the analysis for the long treatment (240 h) indicate that TCSB-AgNPs, especially at the lowest concentration, had no effect in comparison to the control on the parameters of both fungal species tested. In contrast, silver ions at the concentration of 10 mg L^−1^ had the strongest toxic effect on mycelial growth, the mycelium growth rate index and sporulation. CHSB-AgNPs at concentrations of 5 and 10 mg L^−1^, TCSB-AgNPs at the highest concentration and silver ions at a concentration of 5 mg L^−1^ also had a negative impact on all parameters of *F. equiseti*, as did CHSB-AgNPs at a concentration of 10 mg L^−1^ relative to *F. avenaceum,* albeit on a smaller scale than the highest concentration of silver ions. The other treatments had a negligible impact or no effect on the developmental parameters of both species of fungi. The mycelium growth parameter was strongly correlated with the mycelium growth rate index (r = 0.931). As in the short treatment, *F. equiseti* conidia treated for 240 h with the compounds showed a stronger reaction than *F. avenaceum* conidia.

## Discussion

In recent years, AgNPs have been intensively applied to control microbial proliferation^36,46,47^. This study assessed the use of AgNPs to control important pathogens from the point of view of crop protection, namely fungi of the genus *Fusarium: F. avenaceum* and *F. equiseti*. It was shown that the investigated negatively charged TCSB-AgNPs and positively charged CHSB-AgNPs are able to reduce these pathogens. It was found that positively charged CHSB-AgNPs stabilized by cysteamine molecules exhibited higher antifungal activity than negatively charged TCSB-AgNPs coated with citrate anions.

Numerous literature reports have shown that the physicochemical properties of AgNPs, including morphology, surface charge and chemistry of stabilizing layers, can be tuned already at the stage of their synthesis. It is well-known that, by selection of biologically active stabilizers of AgNPs, one can induce synergistic effects and enhance the toxicity of whole nanometric systems towards diverse pathogens^33,48^. For instance, Kasemets et al.^49^, proved that positively charged AgNPs coated with branched polyethylenimine (bPEI) were 8–44 times more toxic to unicellular yeast *Saccharomyces cerevisiae* BY4741 compared to negatively charged citrate-stabilized AgNPs. Thereby, our findings related to the toxicity of positively charged CHSB-AgNPs and negatively charged TCSB-AgNPs towards both strains of *Fusarium* are consistent with the results of Kasemets’ study^49^. Our studies showed also a negative effect of the CH stabilizer against *Fusarium* spp. CH limited the growth of *F. equiseti* after 240 h of treatment and sporulation of *F. avenaceum* in both the long and short treatments (24 and 240 h). CH molecules also made it possible to generate a positive charge on the surface of CHSB-AgNPs, which increased its toxicity. Therefore, the hypothesis was confirmed that the biocidal activity of AgNPs will depend on the presence of the stabilizing agent molecules and surface charge generated by these molecules. In the case of the TC stabilizer, it was shown that it had no or little effect on the tested vital parameters of the fungi, which could have resulted in a lower toxicity of TSCB-AgNPs towards the pathogens compared to CHSB-AgNPs. In turn, Kriti et al.^47^ observed significant activity of citrate-stabilized AgNPs, especially at a concentration of 100 mg L^−1^, towards *Bipolaris sorokiniana* and *Alternaria brassicicola* in terms of vegetative mycelium growth and spore germination.

In our study, the fungistatic activity of AgNPs and silver ions differed depending on the *Fusarium* strains. The *F. equiseti* strain showed greater sensitivity to the silver compounds compared to the *F. avenaceum* strain. The observed differences between strains in response to the AgNPs may result from different resistance mechanisms of the tested fungi^50,51^.

In our experiment, longer contact (240 h) of conidia with the silver compounds resulted in weaker growth on the PDA medium and indicates a stronger inactivation of conidia compared to the shorter treatment (24 h). This suggests that the effectiveness of silver compounds also depends on the duration of their use. Tarazona et al.^52^ demonstrated complete inhibition of the mycelium growth of *F. graminearum*, *F. culmorum*, *F. sporotrichioides*, *F. langsethiae*, *F. poae*, *F. proliferatum* and *F. verticillioides* after the longest exposure (20–30 h) to citrate-stabilized AgNP at concentarions of 30 and 45 mg L^−1^. Jo et al.^53^ showed that both silver ions and AgNPs (20–30 nm) applied 3 h before inoculation with *B. sorokiniana* and *Magnaporthe grisea* spores effectively reduced the severity of leaf spot on perennial ryegrass. The efficacy of silver compounds was significantly reduced when they were used 24 h after inoculation, suggesting that direct contact of silver with spores is important in inhibiting their viability and thus limiting the progression of disease. This was confirmed by Lamsal et al.^54^ who assessed the effect of AgNPs (7–25 nm) on powdery mildew in cucumbers and pumpkins. AgNPs at concentrations of 10, 30 and 50 mg L^−1^ applied about 3–4 weeks before the outbreak of the disease were much more effective than their application after the appearance of disease symptoms on the plants. Lamsal et al.^54^ established that only the use of AgNPs at a concentration of 100 mg L^−1^ significantly inhibited powdery mildew on both plants both before and after the outbreak of the disease, suggesting that the reduction of phytopathogens causing these diseases depends on the treatment time and AgNP concentration. The same relationship was noted by Carvalho et al.^55^ who proved that AgNPs, copper nanoparticles (CuNPs), manganese nanoparticles (MnNPs), zinc nanoparticles (ZnNPs) and the Priori-Xtra fungicide (200 g L^−1^ azoxystrobin + 80 g L^−1^ cyproconazoles), especially in the highest concentration (500 mg L^−1^), limited the germination of *Cercospora coffeicola* spores. In addition, all NPs, except AgNPs, and the fungicide reduced the mycelial growth rate by approximately 100% compared to the control. In contrast, only these AgNPs and a fungicide limited the severity of the brown eye spot in coffee seedlings of the Mundo Novo 376/4 cultivar, suggesting that these AgNPs are more effective when administered prior to the onset of disease symptoms^55^. Malandrakis et al.^56^ also demonstrated the potential of AgNPs (< 100 nm) as protective fungicides in the early stages of disease initiation by inhibiting spore germination of plant pathogenic fungi. AgNPs were more toxic at the spore germination level of important plant pathogens (*Botrytis cinerea*, *Alternaria alternata*, *Monilinia fructicola*, *Colletotrichum gloesosporioides*, *Verticillium dahliae*) than during mycelial growth and in most cases more effective than the commercial Copperblau-N fungicide containing copper (II) hydroxide (Cu(OH)_2_). In addition, the treatment with AgNPs resulted in a significant reduction in gray mold symptoms caused by *B. cinerea* at 85 and 100% at 100 and 1000 µg mL^−1^, respectively, making them excellent candidates for alternative fungicides against pathogenic fungi. The enhanced toxic effect of NPs on fungal spores compared to the growth of hyphae may result from differences in the structure. The hyphae cell walls are made of chitin (about 20%). In general, the walls of the spores contain less chitin than the hyphae, which makes them more susceptible to heavy metals^57^. Moreover, during the spore germination process, disulfide reductases and glucanases soften the cell walls in order to facilitate the elongation of the germinal sprouts, which creates a sensitive place for toxic substances in contact with the fungal cell^58^.

Numerous studies have shown that higher concentrations of AgNPs may result in greater toxicity in cells^31,59^. This thesis was confirmed also in our experiment by proving that both AgNPs were most active against the tested strains at the highest concentration used, i.e. 10 mg L^−1^. A similar tendency was described by Xia et al.^36^ who confirmed the strong fungistatic effect of AgNPs against *Trichosporon asahii* after using higher concentrations. AgNPs at the dose of less than 2 mg mL^−1^ did not limit colony growth, while concentrations in excess of 8 mg mL^−1^ inhibited their growth. The results of Kim et al.^60^ are consistent with results described previously^36^ in terms of concentration. The authors showed the growth of the pathogenic saccharides *Raffaelea* sp. to be inhibited, especially at higher concentrations (10 and 25 mg L^−1^) of AgNPs. Moreover, AgNPs had a detrimental effect on fungal hyphae and conidia germination. Mahdizadeh et al.^51^ also showed that the use of an appropriate dose of AgNPs can control phytopathogenic species. The species most sensitive to AgNPs were *Phytium aphanidermatum* and *Macrophomina phaseolina*, because their growth was inhibited at all concentrations (6, 8, 10, 12, 14 and 16 mg L^−1^). Another sensitive fungus was *Sclerotinia sclerotiorum*, which was completely inhibited at concentrations higher than 6 mg L^−1^. The growth of *Rhizoctonia solani* AG4 was limited to 90% in concentrations up to 10 mg L^−1^, while the remaining concentrations caused 100% inhibition. In turn, the growth of *R. solani* AG1 was reduced by 75% and 80% after the use of AgNPs at concentrations of 6 and 8 mg L^−1^ respectively, while at concentrations of 10, 12 and 14 mg L^−1^ of AgNPs, the growth of fungi was inhibited by 90%, and the highest concentration of 16 mg L^−1^ caused 100% inhibition. The fungistatic effect of AgNPs against phytopathogens may also apply to fungi useful in agrocenoses, which would be undesirable. However, testing of the same AgNPs against the plant growth-promoting fungus *Trichoderma harzianum* showed a different reaction^51^. The growth limitation of *T. harzianum* was significantly lower (80%, 84% and 90% at concentrations of 6, 8 and 10 mg L^−1^ (respectively) than that of the tested phytopathogens. A negative effect was found only at the highest concentrations (12, 14 and 16 mg L^−1^).

The attachment of AgNPs to the cell membranes of microorganisms may be an initial toxicity-inducing process as it increases the exposure of microorganisms to silver in the ionic form^31,35^. Many scientists believe that AgNPs are highly reactive because they release silver ions, which increases their cytotoxicity inside the cell. This mechanism was referred to as the "Trojan horse type mechanism"^33,61^. Szaniawski et al.^62^ relates the sensitivity of fungi to AgNPs and copper nanoparticles (CuNPs) with the structure and chemical composition of the cell wall. *Phytophthora cactorum* 351.13, belonging to the *Oomycota* type and characterized by the presence of cellulose in the cell wall, showed a complete insensitivity to all AgNPs and CuNPs concentrations (5, 15, 25 and 35 mg L^−1^). The remaining species, i.e. *F. oxysporum* 103, *F. redolens* 229, *Giberella* sp. 168, *Rhizoctonia solani* 5648.01 and 1195.00, *Hebeloma crustuliniforme* W40 and 111/08, belonging to the types *Ascomycota* or *Basidiomycota*, with chitin in the cell wall, showed greater sensitivity to both NPs. AgNPs caused stronger growth inhibition mycelium than CuNPs. The cell wall acts as a barrier against biotic and abiotic stresses and influences the movement of particles between the external environment and the cell. According to Navarro et al.^63^, the fungal cell wall consists of carbohydrates that create a rigid and elusive structure. The main component of the fungal cell is chitin, which is semi-permeable, allowing the small AgNPs to pass through, while restricting the passage of the larger ones. AgNPs and silver ions also reduce or completely inhibit the fatty acid content that plays an important role in cell membrane formation^64,65^. As a result of the action of AgNPs, "holes" are formed on the surface of the cell wall, which cause pore formation, leakage of cytoplasmic content and subsequent cell death^66,67^. In our study, TEM imaging showed that negatively charged TCSB-AgNPs generally deposit on the cell surface. In turn, treatment of *F. avenaceum* with CHSB-AgNPs at a concentration of 10 mg L^−1^ led to unfavorable changes in cells. It has been clearly confirmed that positively charged CHSB-AgNPs attach to the surface of cells, causing local damage to the cell wall, which allows them to penetrate the cell interior. In addition, hardly recognizable organelles and extracellular leakage were observed. Firstly, smaller AgNPs are believed to be more toxic than larger AgNPs because they exhibit a larger active surface and have more reactive surface atoms^40,68,69^. Secondly, smaller AgNPs are more sensitive to oxidative dissolution and, as a result, generate more silver ions, which in turn are considered to be a true reactive toxic agent^70,71^. It should be emphasized that these relationships were also confirmed by the results of our studies. CHSB-AgNPs were characterized by slightly lower size and a higher ion release profile than TCSB-AgNPs (Table S1, Supporting materials) and they exhibited stronger fungicidal properties.

It is worth mentioning that properly selected stabilizers make it possible to tune the electrokinetic properties of AgNPs and, as a consequence, the electrostatic interactions between these nanoparticles and charged cell membranes^72,73^. This issue has been described in numerous literature reports. For instance, Silva et al.^38^ have confirmed that positively charged AgNPs coated with branched polyethylenimine (BPEI-AgNP) showed stronger activity against *Escherichia coli* and *Daphnia magna* than negatively charged citrate-coated AgNP (citrate-AgNPs) and polyvinylpyrrolidone-coated AgNP (PVP-AgNPs). This report remains consistent with our findings established for positively charged CHSB-AgNPs and negatively charged TCSB-AgNPs. However, Silva et al.^38^ reported that, at the given concentrations, silver ions were more toxic than all types AgNPs against *E. coli*. In the case *D. magna*, it was established that the toxicity of silver ions and BPEI-AgNP was not significantly different. It seems plausible that the enhanced toxicity of positively charged AgNPs is associated with attractive electrostatic interactions occurring between them and negatively charges membranes of cells. Overall, it is assumed that these forces facilitate the penetration of AgNPs inside cells^74–77^. In turn, silver ions leached inside cells can easily bind to thiol moieties of proteins and cause protein denaturation^36,78^. The research of Morones et al.^79^ and Du et al.^80^ has revealed that AgNPs also damage the transport system, causing the outflow of intracellular ions, which leads to disruption of cellular processes, i.e. metabolism and respiration. Moreover, independently of physicochemical properties, each type of AgNP generates the formation of reactive oxygen species (ROS). Free radicals can cause lipid peroxidation, resulting in an increase in superoxide dismutase activity (SOD), damage to the integrity of cell membranes, and cell apoptosis^81–83^. Pietrzak et al.^84^ also showed many changes in *Penicillium chrysogenum* cells after the application of AgNPs. The authors observed hyphae shortening and condensation, increased vacuolization, collapsed cytoplasm, disintegration of organelles, nuclear deformation, and fragmentation of chromatin. Similar results have been reported by Xia et al.^36^ who proved that the organelles such as mitochondria, chromatin and ribosomes of *T. asahii* were significantly damaged by AgNPs.

The results obtained supported by literature review confirmed that the surface chemistry of AgNPs plays an important role in their antifungal efficacy. Based on the results collected, one can state that the positive surface charge and enhanced ion release profile of silver ions increase the fungicidal properties of AgNPs. The main advantage of AgNPs as antimicrobials is their pleiotropic mechanism of action, as a result of which they attack microorganisms in multiple structures at one time. It is for these reasons that AgNPs have potential as a unique replacement for antibiotics, which are beginning to fail^85^. The toxicity of individual AgNPs is not yet precisely known, because it varies widely, and it is not possible to establish a common criterion^27^. The reaction of microorganisms to AgNPs is also an individual feature. There is considerable variation in the scale of resistance to AgNPs between species. Therefore, AgNPs will require a thorough assessment before being applied in practice, so as not to lead to unfavorable disturbances in ecosystems^23,86^.

## Conclusions

The research conducted revealed strong antifungal activity of positively charged CHSB-AgNPs, negatively charged TCSB-AgNPs and silver ions against common phytopathogens *F. avenaceum* and *F. equiseti* under in vitro conditions. The *F. equiseti* strain exhibited greater sensitivity towards the AgNPs and silver ions than the *F. avenaceum* strain. This finding proves that the sensitivity of *Fusarium* fungi to silver compounds is an individual feature of the species. Silver ions delivered in the form of AgNO_3_ at a concentration of 10 mg L^−1^ caused the inhibition of growth and sporulation of *F. equiseti* in the 240 h treatment. The action of AgNPs in some cases was comparable to the silver ions released by AgNO_3_, which is a well-known but dangerous compound for microorganisms. Positively charged CHSB-AgNPs showed a much stronger effect against *Fusarium* fungi over both the shorter and longer treatment times than negatively charged TCSB-AgNPs. The toxicity of CHSB-AgNPs can be attributed to the properties of the stabilizer adsorbed on their surface (CH), which enhances the (positive) surface charge effect and thus increases their direct penetration by the fungal cell. This proves that the inactivation of pathogens by AgNPs depends on their surface properties. Based on the results of studies, one can state that CHSB-AgNPs, especially at a concentration of 10 mg L^−1^, may be a suitable alternative to disease management fungicides in agricultural applications.

## Supplementary Information

Supplementary Information.

## Data Availability

The data that support this study will be shared upon reasonable request to the corresponding author.

## Abbreviations

NPs: Nanoparticles
TC: Trisodium citrate
CH: Cysteamine hydrochloride
AgNO_3_: Silver nitrate
AgNPs: Silver nanoparticles
TCSB-AgNPs: AgNPs prepared with the use of sodium borohydride (SB) and trisodium citrate (TC)
CHSB-AgNPs: AgNPs prepared with the use of sodium borohydride (SB) and cysteamine hydrochloride (CH)
