# Supplementary material for: Surface properties-dependent antifungal activity of silver nanoparticles
Source: Sci Rep. 2022 Oct 27;12:18046. doi: 10.1038/s41598-022-22659-2 (PMC9613916; doi:10.1038/s41598-022-22659-2)
Supplement: Supplementary file 1 — Supplementary Information. [file 41598_2022_22659_MOESM1_ESM.docx]

**Supporting materials**

**Surface properties-dependent antifungal activity of silver nanoparticles**

Ewelina Matras^1*^, Anna Gorczyca^1^, Sebastian Wojciech Przemieniecki^2^, Magdalena Oćwieja^3^

^1^Department of Microbiology and Biomonitoring, Faculty of Agriculture and Economics, University of Agriculture in Kraków, Mickiewicz Ave. 21, 31-120 Kraków, Poland, e-mail: ewelina.matras@urk.edu.pl, anna.gorczyca@urk.edu.pl; ORCID: **0000-0003-4293-2482**, 0000-0002-9431-3870

^2^Department of Entomology, Phytopathology and Molecular Diagnostics, University of Warmia and Mazury in Olsztyn, Prawocheńskiego 17, 10-720 Olsztyn, Poland, e-mail: sebastian.przemieniecki@uwm.edu.pl; ORCID: **0000-0003-1222-0310**

^3^Jerzy Haber Institute of Catalysis and Surface Chemistry, Polish Academy of Sciences, Niezapominajek 8, PL-30239 Kraków, Poland, e-mail: magdalena.ocwieja@ikifp.edu.pl; ORCID: **0000-0002-7976-135X**

*Corresponding author:

Ewelina Matras

e-mail: ewelina.matras@urk.edu.pl

Postal address: Department of Microbiology and Biomonitoring, University of Agriculture in Krakow, Mickiewicz Ave. 21 / room 426, PL-31120 Krakow, Poland

| **a)** | | **b)** | |  |
| --- | --- | --- | --- | --- |
|  |  | |  |  |
| **c)** | | **d)** | |  |
|  | |  | | |

**Fig. S1** Typical TEM micrographs (scale bar 100 nm, magnification x200 000) presenting
**a)** TCSB-AgNPs and **b)** CHSB-AgNPs and the size distribution of **c)** TCSB-AgNPs and
**d)** CHSB-AgNPs determined based on TEM imaging.

**Table S1** Chosen physicochemical properties of AgNPs and their stock suspensions.

| **Property [unit]** | **TCSB-AgNPs** | **CHSB-AgNPs** |
| --- | --- | --- |
| compounds used for the AgNPs preparation | trisodium citrate (TC)  sodium borohydride (SB) | cysteamine hydrochloride (CH)  sodium borohydride (SB) |
| mass concentration of AgNPs in the stock suspension [mg L^-1^] | 123 | 136 |
| conductivity of stock AgNPs suspension [μS cm^-1^] | 5.7 | 3.2 |
| pH of stock suspension | 6.2 | 5.9 |
| plasmon absorption maximum [nm] | 394 | 402 |
| size of AgNPs [nm] determined based on TEM | 15±4 | 12±4 |
| diffusion coefficient of AgNPs [ x 10^-7^ cm^2^s^-1^] at temperature of 20^o^C | 2.68±0.2 | 3.31±0.2 |
| hydrodynamic diameter of AgNPs [nm] | 16±5 | 13±3 |
| polidispersity index (PdI) | 0.31 | 0.26 |
| electrophoretic mobility [μmcm (Vs)^-1^] at temperature of 20^o^C | -3.15±0.29 | 2.85±0.14 |
| zeta potential [mV] at temperature of 20^o^C | -66±3 | 58±2 |
| concentration of leached silver ions from the AgNPs dispersed in 10 mg L^-1^ suspensions of DO concentration 6.9 – 7.2 mg L^−1^ and at temperature of 20^o^C after:  0 day (after the suspension purification)  7 days  15 days  30 days | 0  0.47±0.09  0.69±0.23  0.82±0.15 | 0  1.19±0.19  3.15±0.18  3.57±0.12 |

**Table S2** The impact of silver ions, AgNPs and stabilizing agents on the mycelial growth of *F. avenaceum* over 24 h (a) and 240 h (b) of the exposure period. Values marked with the same letters (lowercase letters for the interaction effect as well as the main effects of time and treatment; capital letters for interaction effect separately for the each hour) are not significantly different at p ≤ 0.05. The number given for each treatment represent the concentration expressed in mg L^–1^.

a)

| **Treatment** | **Time [h]** | | | | | | | **Means for treatments** |
| --- | --- | --- | --- | --- | --- | --- | --- | --- |
|  | **24** | **48** | **72** | **96** | **120** | **144** | **168** |  |
| Control | 14.4 x-z ^DE^ | 23.6 q-z ^B-D^ | 33.7 j-z ^AB^ | 42.1 c-t ^BC^ | 49.5 a-n ^B^ | 64.9 a-g ^BD^ | 71.7 a-c ^A-D^ | 42.8 c-e |
| Silver ions 2.5 | 14.7 w-z ^C-E^ | 23.1 r-z ^CD^ | 32.5 l-z ^B^ | 40.7 e-v ^BC^ | 48.7 a-o ^BC^ | 63.9 a-g ^BD^ | 71.4 a-c ^A-D^ | 42.1 e |
| Silver ions 5 | 0 z ^F^ | 0 z ^F^ | 26.2 n-z ^C^ | 37.8 f-w ^D^ | 46.2 a-q ^CD^ | 61.7 a-j ^CD^ | 67.8 a-d ^CD^ | 34.2 g |
| Silver ions 10 | 0 z ^F^ | 0 z ^F^ | 23.6 q-z ^C^ | 33.7 k-z ^E^ | 44.4 b-r ^D^ | 61.5 a-i ^D^ | 67.7 a-d ^D^ | 33.0 h |
| TCSB-AgNPs 2.5 | 16.5 u-z ^A^ | 26.8 m-z ^A^ | 36.4 g-x ^A^ | 44.8 a-q ^A^ | 53.4 a-k ^A^ | 68.7 a-d ^A^ | 75.4 a ^A^ | 46.0 a |
| TCSB-AgNPs 5 | 16.2 v-z ^A^ | 24.9 o-z ^B^ | 34 i-z ^AB^ | 43.1 b-s ^AB^ | 50.6 a-l ^AB^ | 66.7 a-d ^AB^ | 73.5 ab ^AB^ | 44.1 b |
| TCSB-AgNPs 10 | 14 yz ^E^ | 24.4 p-z ^BC^ | 34.5 h-y ^AB^ | 42.3 c-t ^BC^ | 50.2 a-l ^B^ | 65.3 a-f ^AB^ | 72.1 ab ^A-C^ | 43.3 b-d |
| CHSB-AgNPs 2.5 | 16 v-z ^AB^ | 23.7 q-z ^B-D^ | 33.7 j-z ^AB^ | 42.3 c-t ^BC^ | 50.3 a-l ^B^ | 66.1 a-e ^AB^ | 72.6 ab ^AB^ | 43.5 b-d |
| CHSB-AgNPs 5 | 15.7 v-z ^A-C^ | 22.4 s-z ^D^ | 32.7 l-z ^AB^ | 42.2 c-t ^BC^ | 49.9 a-m ^B^ | 65.1 a-f ^BC^ | 71.3 a-c ^A-D^ | 42.8 de |
| CHSB-AgNPs 10 | 0 z ^F^ | 19.2 t-z ^E^ | 32.1 l-z ^B^ | 40.6 e-v ^C^ | 48.2 a-p ^BC^ | 63.3 a-h ^BD^ | 69.7 a-c ^BD^ | 39.0 f |
| TC 10 | 15.8 v-z ^AB^ | 24.3 p-z ^BC^ | 34.1 h-y ^AB^ | 42.7 b-t ^A-C^ | 50.6 a-l ^AB^ | 66.4 a-e ^AB^ | 73.1 ab ^AB^ | 43.9 bc |
| CH 10 | 15.1 w-z ^B-D^ | 23.6 q-z ^B-D^ | 33.6 j-z ^AB^ | 41.8 d-u ^BC^ | 50 a-l ^B^ | 65.4 a-f ^AB^ | 71.9 a-c ^A-D^ | 43.1 b-e |
| **Means for time** | 12.0 g | 20.0 f | 32.3 e | 41.2 d | 49.3 c | 65.0 b | 72.0 a | - |

b)

| **Treatment** | **Time [h]** | | | | | | | **Means for treatments** |
| --- | --- | --- | --- | --- | --- | --- | --- | --- |
|  | **24** | **48** | **72** | **96** | **120** | **144** | **168** |  |
| Control | 15.3 s-x ^A^ | 23.2 n-x ^A^ | 35.3 g-t ^AB^ | 46 b-n ^A^ | 53.5 a-h ^A^ | 62.1 a-e ^A^ | 71.8 a ^A^ | 43.9 a |
| Silver ions 2.5 | 14 t-x ^A^ | 22.1 o-x ^A^ | 32 k-x ^BC^ | 44 d-p ^A^ | 52.6 a-i ^A^ | 60.6 a-e ^A^ | 69.7 ab ^A^ | 42.1 a-c |
| Silver ions 5 | 12.8 u-x ^A^ | 21.6 p-x ^AB^ | 33 i-x ^AB^ | 42.1 e-q ^A^ | 50.1 a-k ^A^ | 58.3 a-g ^A^ | 67.1 a-d ^A^ | 40.7 c |
| Silver ions 10 | 0 x ^C^ | 0 x ^D^ | 17.8 r-x ^D^ | 34 h-w ^B^ | 34 h-w ^C^ | 34 h-w ^C^ | 34 h-w ^C^ | 22.0 f |
| TCSB-AgNPs 2.5 | 15 s-x ^A^ | 22.8 o-x ^A^ | 35.6 g-t ^A^ | 44.3 d-o ^A^ | 51.3 a-k ^A^ | 60.2 a-e ^A^ | 70 ab ^A^ | 42.7 ab |
| TCSB-AgNPs 5 | 14.3 t-x ^A^ | 23.7 n-x ^A^ | 33.2 h-w ^AB^ | 44.6 d-o ^A^ | 52.5 a-i ^A^ | 61 a-e ^A^ | 71.6 ab ^A^ | 43.0 ab |
| TCSB-AgNPs 10 | 14.1 t-x ^A^ | 21.7 0-x ^AB^ | 33.3 h-w ^AB^ | 45.7 b-n ^A^ | 52.5 a-h ^A^ | 61 a-e ^A^ | 69 ab ^A^ | 42.5 a-c |
| CHSB-AgNPs 2.5 | 15 s-x ^A^ | 23.6 n-x ^A^ | 34.6 g-u ^AB^ | 43.8 d-p ^A^ | 51.6 a-j ^A^ | 59.5 a-f ^A^ | 69.3 ab ^A^ | 42.5 a-c |
| CHSB-AgNPs 5 | 0 x ^C^ | 18.8 q-x ^BC^ | 32.2 j-x ^AB^ | 41.5 e-q ^A^ | 44.2 d-o ^B^ | 48.6 a-l ^B^ | 57.2 a-g ^B^ | 34.6 d |
| CHSB-AgNPs 10 | 0 x ^C^ | 17.6 q-x ^C^ | 28.5 l-x ^C^ | 36.2 f-s ^B^ | 41.8 e-r ^B^ | 47 a-m ^B^ | 52.2 a-j ^B^ | 31.9 e |
| TC 10 | 9.8 v-x ^B^ | 24 m-x ^A^ | 34.3 h-v ^AB^ | 44.6 c-o ^A^ | 52.3 a-i ^A^ | 60.6 a-e ^A^ | 69.6 ab ^A^ | 42.2 a-c |
| CH 10 | 8.1 wx ^B^ | 23.6 n-x ^A^ | 33.8 h-w ^AB^ | 43.3 d-p ^A^ | 51 a-k ^A^ | 59.5 a-f ^A^ | 68.7 a-c ^A^ | 41.1 bc |
| **Means for time** | 9.9 g | 20.2 f | 32.0 e | 43.0 d | 49.0 c | 56.0 b | 64.2 a | - |

**Table S3** The impact of silver ions, AgNPs and stabilizing agents on the mycelial growth of *F. equiseti* over 24 h (a) and 240 h (b) of the exposure period. Values marked with the same letters (lowercase letters for the interaction effect as well as the main effects of time and treatment; capital letters for interaction effect separately for the each hour) are not significantly different at p ≤ 0.05. The number given for each treatment represent the concentration expressed in mg L^–1^.

a)

| **Treatment** | **Time [h]** | | | | | | | **Means for treatments** |
| --- | --- | --- | --- | --- | --- | --- | --- | --- |
|  | **24** | **48** | **72** | **96** | **120** | **144** | **168** |  |
| Control | 16.5 r-y ^AB^ | 23.7 o-y ^AB^ | 35.2 j-w ^AB^ | 43.3 c-p ^A^ | 51.6 a-k ^AB^ | 67.4 a-e ^A^ | 75.8 a ^A^ | 44.8 a |
| Silver ions 2.5 | 13.7 v-y ^D^ | 23.8 o-y ^AB^ | 36.9 i-v ^A^ | 46.4 a-n ^A^ | 52.6 a-j ^A^ | 61.4 a-h ^AB^ | 68.8 a-e ^AB^ | 43.4 a |
| Silver ions 5 | 0 y ^E^ | 23.5 p-y ^AB^ | 30.8 m-y ^C^ | 35.8 i-w ^C^ | 42.2 e-q ^BC^ | 51.3 a-l ^BC^ | 56.5 a-i ^BC^ | 34.3 b |
| Silver ions 10 | 0 y ^E^ | 0 y ^E^ | 2.1 xy ^D^ | 14.5 s-y ^D^ | 23.8 n-y ^D^ | 36.2 g-t ^D^ | 41.5 c-p ^C^ | 16.9 d |
| TCSB-AgNPs 2.5 | 15.2 t-y ^B-D^ | 25 n-y ^A^ | 36.1 i-v ^AB^ | 44.6 a-n ^A^ | 52 a-j ^A^ | 66.9 a-e ^A^ | 74.5 a ^A^ | 44.9 a |
| TCSB-AgNPs 5 | 15.2 t-y ^B-D^ | 24.5 n-y ^AB^ | 34.3 j-x ^A-C^ | 43.3 c-p ^A^ | 51.3 a-l ^AB^ | 65.5 a-f ^AB^ | 72.4 ab ^AB^ | 43.8 a |
| TCSB-AgNPs 10 | 14.3 v-y ^D^ | 19.9 q-y ^C^ | 32.5 k-y ^BC^ | 43.2 e-p ^A^ | 50.4 a-m ^AB^ | 63.9 a-h ^AB^ | 71.7 a-d ^AB^ | 42.3 a |
| CHSB-AgNPs 2.5 | 15.1 u-y ^B-D^ | 24.7 n-y ^A^ | 35.5 i-w ^AB^ | 44.7 b-o ^A^ | 52.8 a-j ^A^ | 64.9 a-g ^AB^ | 71.9 a-c ^AB^ | 44.2 a |
| CHSB-AgNPs 5 | 14.6 v-y ^CD^ | 24.6 n-y ^AB^ | 35.3 j-w ^AB^ | 42.8 e-q ^AB^ | 50.3 a-m ^AB^ | 61 a-h ^AB^ | 66.5 a-e ^AB^ | 42.2 a |
| CHSB-AgNPs 10 | 0 y ^E^ | 12.3 w-y ^D^ | 32 l-y ^BC^ | 36.5 i-v ^BC^ | 38.4 h-u ^C^ | 38.9 g-s ^CD^ | 39.8 f-r ^C^ | 28.3 c |
| TC 10 | 17.2 r-y ^A^ | 24.6 n-y ^AB^ | 35.3 j-w ^AB^ | 43.2 d-p ^A^ | 51.8 a-k ^A^ | 68.6 a-e ^A^ | 74.5 a ^A^ | 45.0 a |
| CH 10 | 16.2 r-y ^A-C^ | 22.5 p-y ^B^ | 34 j-x ^A-C^ | 43 e-p ^A^ | 50.8 a-l ^AB^ | 65.6 a-f ^AB^ | 73.5 ab ^A^ | 43.7 a |
| **Means for time** | 11.5 g | 20.8 f | 31.7 e | 40.1 d | 47.3 c | 59.3 b | 65.6 a | - |

b)

| **Treatment** | **Time [h]** | | | | | | | **Means for treatments** |
| --- | --- | --- | --- | --- | --- | --- | --- | --- |
|  | **24** | **48** | **72** | **96** | **120** | **144** | **168** |  |
| Control | 16.3 t-z ^A^ | 24.5 n-z ^AB^ | 36.6 g-x ^A^ | 47.2 a-n ^A^ | 55.1 a-g ^A^ | 63.5 ab ^A^ | 75.1 a ^A^ | 45.5 a |
| Silver ions 2.5 | 14.6 v-z ^A-C^ | 24.6 n-z ^AB^ | 36.3 g-y ^A^ | 46.2 a-o ^AB^ | 52 a-k ^AB^ | 57.8 a-f ^AB^ | 68 a ^A-C^ | 42.8 ab |
| Silver ions 5 | 13.1 x-z ^C^ | 24.3 o-z ^AB^ | 34.6 i-y ^A^ | 40.6 c-u ^BC^ | 47.8 a-m ^BC^ | 54 a-h ^B^ | 60.8 a-d ^C^ | 39.3 b |
| Silver ions 10 | 0 z ^E^ | 0 z ^G^ | 0 z ^C^ | 0 z ^F^ | 0 z ^E^ | 0 z ^D^ | 0 z ^E^ | 0.0 d |
| TCSB-AgNPs 2.5 | 15.3 u-z ^AB^ | 20.5 q-z ^D^ | 36.2 g-y ^A^ | 45.5 a-p ^A-C^ | 53.3 a-i ^AB^ | 59.6 a-e ^AB^ | 69.2 a ^A-C^ | 42.8 ab |
| TCSB-AgNPs 5 | 14.8 v-z ^A-C^ | 23.1 p-z ^BC^ | 34.8 i-y ^A^ | 45.8 a-p ^AB^ | 52.7 a-j ^AB^ | 59.3 a-e ^AB^ | 68.2 a ^A-C^ | 42.7 ab |
| TCSB-AgNPs 10 | 6.7 yz ^D^ | 17.5 r-z ^E^ | 24.8 n-z ^B^ | 34 j-y ^DE^ | 38.3 e-v ^D^ | 42.3 b-r ^C^ | 48.3 a-m ^D^ | 30.3 c |
| CHSB-AgNPs 2.5 | 14.6 v-z ^A-C^ | 24.3 n-z ^AB^ | 37.3 g-x ^A^ | 46.7 a-o ^A^ | 51.7 a-k ^AB^ | 56.2 a-g ^AB^ | 64.5 ab ^BC^ | 42.2 ab |
| CHSB-AgNPs 5 | 0 z ^E^ | 13.8 x-z ^F^ | 25.6 m-z ^B^ | 39.5 d-u ^CD^ | 41.3 b-t ^CD^ | 42.3 b-r ^C^ | 49.1 a-l ^D^ | 30.2 c |
| CHSB-AgNPs 10 | 0 z ^E^ | 17.2 r-z ^E^ | 24.8 n-z ^B^ | 33.3 k-z ^E^ | 38.1 f-w ^D^ | 42 b-s ^C^ | 44.8 a-p ^D^ | 28.6 c |
| TC 10 | 16.3 s-z ^A^ | 26.5 l-z ^A^ | 37.2 g-x ^A^ | 47.8 a-m ^A^ | 54.6 a-g ^AB^ | 61.8 a-c ^AB^ | 72.5 a ^AB^ | 45.2 a |
| CH 10 | 14.1 w-z ^BC^ | 21.2 q-z ^CD^ | 33.8 h-y ^A^ | 43.5 a-q ^A-C^ | 51.1 a-k ^AB^ | 56.3 a-g ^AB^ | 64.3 ab ^BC^ | 40.6 b |
| **Means for time** | 10.5 g | 19.8 f | 30.2 e | 39.2 d | 44.7 c | 49.6 b | 57.1 a | - |

**Table S4** Spearman correlation for mycelial growth of *Fusarium* spp. after silver ions, AgNPs and stabilizing agents treatments.

| **Results of Spearmen test for *F. avenaceum* vs *F. equiseti*** | | | | | |
| --- | --- | --- | --- | --- | --- |
|  |  |  |  |  | Value |
| Correlation matrix | | | | | 0.920 |
| Coefficient of determination | | | | | 0.847 |
| p-values | | | | | <0.0001 |
